# Congenital heart block associated with Sjögren syndrome: case report

**DOI:** 10.1186/1755-7682-2-21

**Published:** 2009-07-28

**Authors:** Karwan A Moutasim, Penelope J Shirlaw, Michael P Escudier, Timothy WJ Poate

**Affiliations:** 1 Cancer Sciences Division, School of Medicine, University of Southampton, Southampton SO16 6YD, UK; 2 Department of Oral Medicine, Guy's and St. Thomas' NHS Foundation Trust, London SE1 9RT, UK; 3 King's College London Dental Institute at Guy's, King's and St. Thomas' Hospitals, London SE1 9RT, UK; 4 King's College Hospital NHS Foundation Trust, Denmark Hill, London SE5 9RS, UK

## Abstract

**Background:**

Congenital heart block is a rare complication of pregnancy associated with Sjögren Syndrome that may result in the death of the foetus or infant, or the need for pacing in the newborn or at a later stage.

**Case report:**

The case is presented of a 64-year-old patient with primary Sjögren Syndrome and a history of having given birth to two sons with congenital heart block, both of whom required pacing several years later.

**Conclusion:**

The literature relating to this association is discussed including the suggested mechanism, long-term outcome of mothers of children with congenital heart block and preventive treatment strategies.

## Background

Sjögren Syndrome is an autoimmune disease of exocrine glands that occurs with a prevalence of 0.5 – 1% [1]. It has a female preponderance and is commonly diagnosed in the 4^th^–5^th ^decades of life. As it primarily affects the lachrymal and salivary glands, the chief clinical features are dry eyes and a dry mouth [2].

Congenital heart block occurs in a frequency of 1 in 20,000 live births. It has been reported to occur in 2% of Ro-positive mothers [3]; 5% of mothers with a diagnosis of mixed connective tissue and/or Sjögren Syndrome [4] and in 8% of Ro-positive mothers [5]. 53% of cases are diagnosed at 16–24 weeks of gestation; whilst 24% are diagnosed later at 25–30 weeks of gestation [6]. Here, we present the case of a female patient diagnosed with Sjogren Syndrome that had given birth to two sons with congenital heart block with different presentation and management.

## Case Report

A 64-year-old Caucasian lady with Sjögren Syndrome was referred to the Oral Medicine department from Rheumatology. She complained of dry mouth, eyes and vagina; intermittent swelling of the salivary glands and aching joints. She was diagnosed with primary Sjögren Syndrome 35 years earlier. At presentation, her management regime consisted of 5 mg prednisolone, Oral Balance Gel, sugar-free chewing gum and Viscotears. Her other medications were lansoprazole 15 mg, hydroxychloroquine 200 mg and thyroxine 75 mcg daily. She was a retired journalist. She did not smoke. She drank 14 units of alcohol a week, mainly red wine. She was married with 5 sons; 2 of whom had congenital heart block. The first son was 41 years old and had required no treatment. The second son was 25 years old and had required a pacemarker at age 4 years and later required a heart transplant at 13 due to cardiomyopathy. Extraoral examination revealed mild skin bruising. Intraorally, the mucosa was dry with no salivary pooling and no milkable saliva from the major gland orifices. Blood investigations revealed lymphopenia at 0.9 × 10^9 ^(reference range: 1.3–3.5 × 10^9^), a raised ESR of 38 (reference range: 1–15) and a raised CRP of 8 mg/L (reference range:0–4 mg/L). She was anti-Ro and anti-La positive and IgG levels were raised at 26.2 (5.3–16.5 g/L). Her whole flow rate was 0 ml/min and the parotid flow rate was markedly low at <0.1 ml/min. *C. albicans *counts were raised. Her management was based on maintaining good hydration. The superimposed candidal infection was treated by 1-week courses of Nystatin 100,000 IU/ml oral suspension, repeated every 4 weeks.

## Discussion and Conclusion

An association between congenital heart block (CHB) and connective tissue diseases such as lupus and Sjögren Syndrome has been reported in the literature [7]. There is a strong correlation between congenital heart block and maternal autoantibodies to 48 kD La, 52 kD Ro and 60 kD Ro ribonucleoproteins. The resultant fibrosis of foetal AV node is thought to be due to immune mediated tissue damage to foetal heart following transplacental passage of maternal IgG autoantibodies [8].

A study by Buyon et al examined 105 mothers with Ro and/or La positive sera, and reported that 113 infants were diagnosed with CHB. Of the 87 pregnancies with adequate records, bradyarrhythmias detected before 30 weeks gestation in 71 (82%) – median 23 weeks. Of the 113 infants, 22 (19%) died, mostly within 3 months of birth. Of the 107 live-born children, 67 (63%) required pacing; 35 within 9 days, 15 within 1 year and 17 after 1 year. Of the 49 mothers who had subsequent pregnancies, 8 (16%) had another child with CHB [6].

The underlying mechanism behind congenital heart block in Sjögren Syndrome is suggested to be due to Ro and La antigens present on apoptotic blebs on surface of actively remodelling foetal cardiocytes. Opsonized Ro & La antibodies can then lead to macrophage activation, TNF-α & TGF-β secretion which favours differentiation of fibroblasts into collagen-secreting myofibroblasts which promote scarring [8]. The site of action of Ro & La antibodies may be the α-1 subunit of the L-type calcium channel, which is involved in action potential propagation in AV node & excitation coupling in the heart. These channels are present in lower density in foetal heart cells; which may explain why the foetal heart is affected and the maternal heart unaffected [7,8].

It is thought that foetal CHB arises due to increased susceptibility of the foetal heart due to increased apoptotic remodelling of foetal heart cells or lower density of L-type calcium channels. Other antibodies such as muscarinic type-1 receptor (M1R) antibodies described in maternal blood may also have a role. Foetal heart expresses M1R while maternal heart expresses mainly M2R. The majority of cases of CHB are subclinical at birth; with a case reported of normal ECG at birth and subsequent development of asymptomatic heart block at 2 years [9].

Discordance for CHB has been reported in monozygotic twins despite identical genetics and environmental exposure to maternal anti-Ro antibody [10]. Late-onset dilated cardiomyopathy has been reported despite early pacing in 16 children with congenital heart block [11]. Dilated cardiomyopathy and heart failure developed between 2 weeks and 9 years requiring cardiac transplant in those who survived.

It is recommended that infants with CHB require close follow up, not only of cardiac rate & rhythm, but also of ventricular function. Press *et al *studied the long-term outcome of 64 children with complete heart block, and found that 60% (32 out of 53) of the mothers tested were anti-Ro and/or anti-La positive. The mean follow-up period was 121 months, and the mean maternal age was 38. At the time of delivery, the majority of mothers (66%) were healthy [12].

Potentially serious outcomes are rare but possible, with 63% of affected offspring requiring pacing. The general recommendation is to monitor foetal cardiac function from 16–24 weeks *in utero*, continuing after birth to monitor rate, rhythm and ventricular function [7].

The optimal treatment is still debatable but corticosteroids may be of benefit in established cases and may further have a role in the prevention of congenital heart block in mothers with previously affected pregnancy. Thus, treatment of congenital heart block is based on fluorinated steroids that cross the placenta. The use of glucocorticoid intervention for 3–19 weeks in cases of foetal second degree heart block may increase the likelihood of reverting to first degree heart block rather than progression to third degree heart block [13]. Dexamethasone commenced at time of diagnosis of CHB and maintained for duration of pregnancy may also improve foetal survival in cases of prenatally diagnosed complete heart block [14].

The evidence to support prophylactic treatment to prevent congenital heart block during subsequent pregnancies is weak, and concerns regarding the safety of fluorinated steroids have been raised, particularly neurological toxicity, spontaneous abortion, stillbirths & intrauterine growth restriction. Other interventions, such as intravenous immunoglobulins, plasmapeheresis and azathioprine have been suggested [15].

In summary, congenital heart block is a common occurrence in offspring of mothers affected with Sjögren Syndrome. Whilst there is no consensus on management, appropriate referral and follow-up is recommended. Finally, a diagnosis of Sjögren Syndrome should be considered in a mother who has a child diagnosed with congenital heart block.

## Consent

Written informed consent was obtained from the patient as per the standard protocol at Guy's Hospital NHS Foundation Trust.

## Competing interests

The authors declare that they have no competing interests.

## Authors' contributions

All authors were involved in patient diagnosis and management, with PJS and MPE being the senior clinicians guiding overall management at the time. KAM wrote the manuscript under the general guidance and supervision of TWJP.

## Authors Information

K.A.M., BDS, MFDRCSI, MSc is currently a Clinical Research Training Fellow at the University of Southampton. Prior to that, he had been a postgraduate student and research fellow at Guy's Hospital (the study site). He is a member of the British Society for Oral Medicine, British Society for Oral and Maxillofacial Pathology, International Association for Oral Pathology and the International Association for Dental Research. He has won numerous clinical and research prizes including the Royal Society of Medicine's President's Prize of the Odontology Section (2009; UK) and the British Society for Oral Medicine's clinical and research papers prize (2009).

P.J.S., BDS, LDSRCS, FDSRCS, is a Consultant in Oral Medicine and the Clinical Director for Dental Services at Guy's Hospital, London (UK).

M.E.P. BDS, MBBS, FDSRCS, MRCP, FDSRCS(OM), MD is a Senior Lecturer and Honorary Consultant in Oral Medicine at Guy's Hospital (UK).

T.W.J.P. BDS, MBBS, FDSRCS, MRCP, FDSRCS(OM) is currently a Consultant in Oral Medicine at King's College Hospital, London. He has trained in both dentistry and medicine and at the time of the study was a Specialist Registrar and Honorary Lecturer in Oral Medicine.
